# Genomic Analysis Reveals Inbreeding in an Island Population of Alexander Archipelago Wolves

**DOI:** 10.1111/eva.70144

**Published:** 2025-08-12

**Authors:** Katherine E. Zarn, Gretchen H. Roffler, Marty Kardos, Jeffrey M. Good, Daniel Vanderpool, Taylor Wilcox, Michael K. Schwartz

**Affiliations:** ^1^ National Genomics Center for Wildlife and Fish Conservation, Rocky Mountain Research Station U.S. Department of Agriculture, Forest Service Missoula Montana USA; ^2^ Division of Wildlife Conservation Alaska Department of Fish and Game Juneau Alaska USA; ^3^ Northwest Fisheries Science Center National Oceanic and Atmospheric Administration Seattle Washington USA; ^4^ Flathead Lake Biological Station University of Montana Polson Montana USA; ^5^ Division of Biological Sciences University of Montana Missoula Montana USA

**Keywords:** *Canis lupus*, genomic monitoring, island populations, landscape fragmentation, runs of homozygosity, sequence capture array

## Abstract

Island populations are at heightened risk of inbreeding due to reduced mating opportunities with unrelated conspecifics. Extensive inbreeding can result in inbreeding depression (reduced fitness of individuals with related parents). Alexander Archipelago wolves ( *Canis lupus ligoni* ) are a geographically isolated subspecies that occur in the Southeast Alaskan panhandle, USA, and coastal British Columbia, Canada. Wolves on the Prince of Wales Island complex (POW) in Southeast Alaska are expected to have lower levels of resiliency because they are a small, insular population that has experienced habitat fragmentation and cycles of moderate to heavy harvest. To understand the extent of population structure and inbreeding in Alexander Archipelago wolves, we designed a DNA hybridization capture for wolves and sequenced captured DNA from 58 individuals sampled from across Southeast Alaska during 2002–2016. Estimates of the proportion of the genome in runs of homozygosity (*F*_ROH_) regardless of run length, revealed that POW wolves were most inbred compared to wolves in other areas of Southeast Alaska. Wolves on POW also had more long (≥ 10 Mb) runs of homozygosity than the other populations we assessed, indicating more frequent mating between individuals with recent common ancestors (1–10 generations ago). This pattern indicates a smaller population size for POW wolves in the recent past compared to other Southeast Alaskan populations. Wolves on POW exhibit an extent of inbreeding similar to that observed in Isle Royale National Park wolves, a population that has exhibited severe inbreeding depression. Our work demonstrates the utility of using genomic capture data to infer individual inbreeding so that proactive management (e.g., setting population targets and harvest quotas, curtailing habitat alteration, etc.) can be considered to ensure the long‐term sustainability of small, isolated populations.

## 1 Introduction

Habitat loss, climate change, and other anthropogenic pressures are increasingly causing wildlife populations to become smaller, fragmented, and isolated, which can result in a cascade of negative outcomes (Shaffer 1987; Keller and Waller 2002; Lobo et al. 2023). One such consequence is an increase in inbreeding (i.e., mating between relatives). Island populations are at a greater risk of inbreeding than their mainland counterparts because of low genetic diversity, small population size, and geographic isolation, which results in reduced mating opportunities with unrelated conspecifics (Frankham 1998; Colpitts et al. 2022). Inbreeding decreases heterozygosity and standing genetic variation, which reduces the resilience of populations and their potential to adapt to changes in the environment. Importantly, prolonged or extensive inbreeding can result in inbreeding depression (e.g., reduced fitness of individuals with closely related parents) and reduced population viability (Keller and Waller 2002; Charlesworth and Willis 2009). Therefore, the development of effective genomic tools can facilitate wildlife managers in monitoring the extent of inbreeding and implementing mitigation actions that may quell inbreeding depression before it results in a population decline and other deleterious effects (e.g., von Holdt et al. 2024).

Wolves are highly social carnivores living in packs typically comprised of a single breeding male and female and may also include non‐breeding individuals (Mech and Boitani 2003). As territorial, cooperatively breeding canids, behavioral strategies such as dispersal outside of natal groups and avoiding breeding with close kin can alleviate the potential for inbreeding (von Holdt et al. 2008; Geffen et al. 2011; Ausband 2022a). One such documented strategy is for females to remain with their natal group to wait for a breeding opportunity with unrelated immigrant males (Ausband 2022b; Pacheco et al. 2024) although both sexes disperse from their natal packs (Morales‐González et al. 2022). However, in circumstances where dispersal is impeded by geographic or anthropogenic barriers, co‐occurrence and breeding with closely related individuals can occur more frequently and lead to inbreeding and potentially inbreeding depression (Liberg et al. 2005; Hedrick et al. 2014).

**FIGURE 1:**
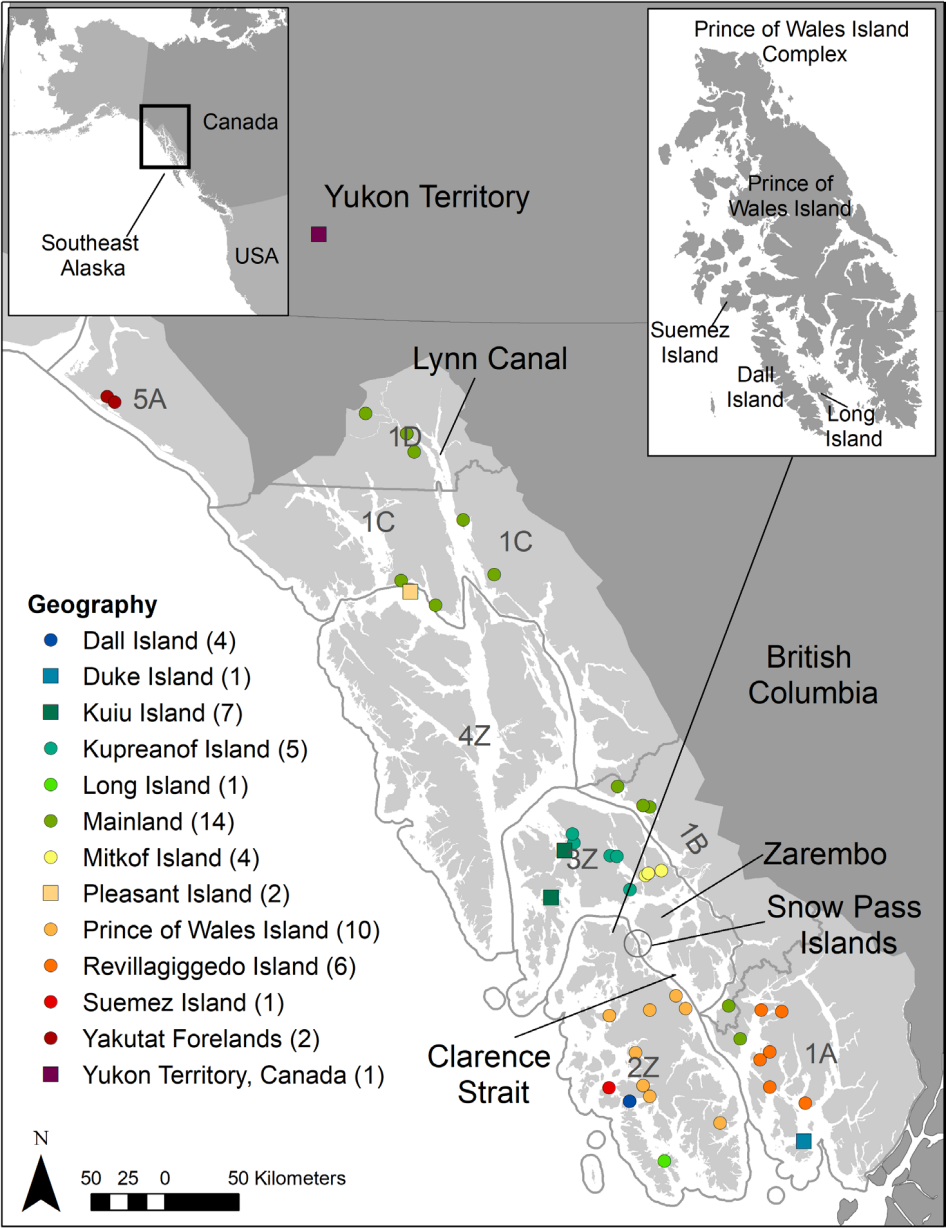
Map of Southeast Alaska, USA and adjacent Canada showing locations and numbers of wolves included in this study (*n* = 58) by sampling location, Game Management Units and Subunits (dark gray text and outlines), and geographical features referenced in main text. Wolves do not inhabit islands in Game Management Unit (GMU) 4Z, with the exception of Pleasant Island which was administratively changed to GMU 1C in 2022.

Alexander Archipelago wolves ( *Canis lupus ligoni* ) on the Prince of Wales Island complex (POW) in Southeast Alaska, USA (Figure 1) present a strong case study for detecting and characterizing inbreeding in a small, geographically isolated population of conservation concern. The Prince of Wales Island complex is designated as Game Management Unit 2 (GMU 2) and managed as a single population. Wolf dispersal among the islands of GMU 2 has been documented through radio and GPS‐collared wolves (Person and Ingle 1995; Roffler et al. 2016) and from recapture and genetic data (Roffler et al. 2024). Wolf dispersal is common and may include long‐distance movements (Mech 2020), even in archipelagic landscapes as wolves are capable of swimming and using smaller islands as stepping‐stones (Darimont and Paquet 2002; Muñoz‐Fuentes et al. 2010; Stronen et al. 2014; Collins et al. 2019). However, of the radio‐collared wolves monitored on POW during 1993–1995, 1999–2004, and 2012–2018 (*n* = 68; Person and Russell 2008; Roffler et al. 2018), none have been documented traveling the three to seven kilometer distance between POW and adjacent islands or to mainland peninsulas in other GMUs (Figure 1). This geographic isolation is reflected by high levels of genetic differentiation of POW in relation to other geographical areas in Southeast Alaska documented in previous genetic analyses using microsatellites and single nucleotide polymorphisms (SNPs; Weckworth et al. 2005; Cronin et al. 2015).

In addition to the presence of geographic barriers to movement and dispersal, old‐growth forests on POW have been heavily logged since the 1950s, resulting in decreased quantity and quality of habitat for both wolves and their main prey, Sitka black‐tailed deer ( *Odocoileus hemionus sitkensis* ; Hanley 1984; Albert and Schoen 2013). Wolves on POW have also experienced periods of moderate to heavy harvest, with annual harvest ranging from 7 to 164 wolves since 1985 (Roffler et al. 2024; Figure S1A). The population has been estimated annually, with the lowest recorded abundance in 2014 (*n* = 89, 95% CI = 50–159) and the highest in 2020 (*n* = 386, 95% CI = 321–472) (Dorendorf 2022; Figure S1B). Given the geographic isolation, habitat loss, and harvest pressure, we sought to understand whether wolves on POW were more inbred than wolves elsewhere in Southeast Alaska and whether inbreeding in POW is more strongly driven by recent or long‐term demography.

To assess inbreeding in POW wolves, we required a method that was sensitive enough to detect and differentiate inbreeding that arose due to mating between individuals with more recent or more distant common ancestors. This would allow us to understand whether wolves on POW have experienced low levels of inbreeding over a long period of time or if they have experienced a recent increase in inbreeding, perhaps as the result of anthropogenic pressures on the population. High‐throughput sequencing approaches have greatly improved the accuracy and precision of many approaches in population genomics, including estimates of individual inbreeding using runs of homozygosity (ROH; Kardos et al. 2015). Runs of homozygosity are stretches of the genome that are homozygous and identical‐by‐descent (IBD) due to parental common ancestors (Thompson 2013). Long ROH occur in offspring produced by inbreeding between parents with more recent common ancestors and are broken down into shorter segments over generations by recombination events during meiosis. The progressive shortening of ROH through generations by recombination means that ROH lengths can be used to estimate the coalescent time for the IBD haplotypes (Kardos et al. 2016).

Here we describe the development and application of a high‐resolution targeted genomic sequencing method to infer the extent and timing of inbreeding in POW wolves. To accomplish this goal, we collected tissue samples from wolves (*n* = 58) harvested during 2002–2016 throughout Southeast Alaska, including POW and in adjacent Yukon, Canada. We used a custom hybridization‐based capture to target tens of thousands of non‐coding SNPs. Using the SNP genotypes, we assessed spatial genetic structure and identified populations within the sampled region. Finally, we conducted ROH analysis to assess levels of inbreeding and compare likely histories of inbreeding across these putative populations. We included previously sequenced genomic data from Isle Royale National Park (IRNP; Robinson et al. 2019) to make comparisons to a well‐studied island population of wolves that experienced severe inbreeding depression.

Data to inform inbreeding depression are not currently available for Alexander Archipelago wolves in southeast Alaska. However, contextualizing the extent of genomic inbreeding in Southeast Alaskan wolves to the extent of genomic inbreeding observed in IRNP wolves provides an important platform to support future data collection and work to evaluate the potential for inbreeding depression in Southeast Alaskan wolves. It is possible that long‐term isolation of wolves in Southeast Alaska has resulted in the purging of strongly deleterious recessive alleles, resulting in a small and isolated but stable population, as has been observed in other taxa (Robinson et al. 2023). In this scenario, the high extents of inbreeding reported here may not be indicative of inbreeding depression and a potential source of instability for long‐term persistence of the population. Our results show a high extent of inbreeding in a geographically isolated population, providing critical information to support future studies on inbreeding depression and to facilitate management and conservation in this system.

## 2 Materials and Methods

### 2.1 Study System

Southeast Alaska is an expansive archipelagic landscape with over 2000 named islands and includes a narrow strip of mainland bordered to the east by the heavily glaciated Coast Mountains (MacDonald and Cook 2007). These landscape features and geographic isolation have limited gene flow with neighboring continental wolf populations (Weckworth et al. 2005; Pacheco et al. 2022). Prince of Wales Island is located in the southern portion of Southeast Alaska (Figure 1) and is the fourth‐largest island in the United States, covering 6670 km^2^ with 1593 km of coastline characterized by numerous bays, inlets, and fjords. The Prince of Wales Island complex includes an additional 25 islands ranging from 5 to 661 km^2^ and hundreds of smaller islands. The landscape of POW contains lakes, streams, river valleys, and mountainous terrain up to 1,160 m in elevation. The vegetative communities on POW include old‐growth conifer forests, post‐clear‐cut forest stands of uniform ages at varying successional stages and, in less abundance, freshwater muskeg bogs and riparian and alpine areas. In addition to wolves, POW also supports populations of other mammals such as Sitka black‐tailed deer, North American beaver ( *Castor canadensis* ), North American river otter ( *Lontra canadensis* ), American marten ( *Martes americana* ), and black bear ( *Ursus americanus* ). Extensive old‐growth logging has resulted in a 94% reduction of contiguous high‐volume forests between 1954 and 2004 on northern POW (Albert and Schoen 2013) and old‐growth timber sales and logging continue into the present day. Roads built for timber harvest also provide increased access for hunters and trappers, facilitating high wolf harvest (Person and Russell 2008).

### 2.2 Sample Collection and Preparation

Samples for this study were obtained opportunistically from a larger sampling effort undertaken by the Alaska Department of Fish and Game (ADF&G) to survey population genetic structure and connectivity of wolves in Southeast Alaska. Tissue samples were collected during 2002–2016 (Table S1) from all GMUs in Southeast Alaska where wolves occur, including on Pleasant Island, which was formerly part of GMU 4Z until 2022 when it was changed to be a part of GMU 1C because of its closer geographical connection to the northern mainland. We also included 1 sample from the adjacent Yukon Territory, Canada, for comparison to interior wolves. Samples were collected in accordance with guidelines established by ADF&G Animal Care and Use Committee (ACUC #2012–028 and #2014–15) and the American Society of Mammologists (Sikes and Gannon 2011) or by voluntary tissue sample submission to ADF&G from trapper and hunter harvest. We extracted DNA from tissue samples using the DNeasy Blood and Tissue Kit (Qiagen) following the manufacturer's protocol, substituting buffer AE with TET buffer (10 mM Tris–HCl, 1 mM EDTA, 0.05% Tween‐20, pH 8.0).

### 2.3 Capture Design and Optimization

Wildlife conservation and management resources are limited, and we sought to develop a cost‐effective genotyping method that would maximize sample size to assess genomic inbreeding and population genetic structure in a population of conservation concern. We also sought to develop a method that would induce less ascertainment bias than commercially available canine SNP genotyping methods. Commercially available SNP genotyping methods that are compatible with wolves are potentially cost‐effective options, but are typically developed using a small number of domestic dogs to identify variable sites and with the intent to conduct breed association studies and/or evaluate genetic risk factors (e.g., Illumina CanineSNP20 Panel, Broad Institute Canine Array v1 and v2, etc.) The demographic history of dogs includes significant population bottlenecks, maintenance of small effective population sizes (breeds), and inbreeding, which has resulted in domestic dogs carrying relatively little genetic variation when compared to wild canids (Freedman et al. 2014; Marsden et al. 2016). Domestic dogs, particularly purebred dogs, also exhibit increased instances of recessive genetic disorders not commonly observed in wild canids (e.g., Pedersen et al. 2015; Leroy 2011; Farrell et al. 2015; Oberbauer et al. 2015; Marsden et al. 2016; Axelsson et al. 2021). SNP panels designed using a small number of dogs with the intent to perform breed association and genetic disease studies are biased in favor of identifying variation specific to dog breeds and genetic disorders and could therefore carry significant ascertainment bias when applied to population genetic applications in wild wolves. We developed a cost‐effective method that is less likely to incur ascertainment bias and more likely to reflect true population structure when applied to wild wolf populations.

We used the domestic dog reference genome (canFam3.1; Lindblad‐Toh et al. 2005) to design a hybridization capture probe targeting 100,144,500‐bp regions spaced ≥ 19,700 nucleotides apart. Hybridization‐based capture will enrich target DNA that is up to 40% divergent, and although enrichment efficiency begins to decline at 4%–10% divergence (Paijmans et al. 2016), genome‐wide pairwise sequence divergence between dogs and wolves is ~0.11% on average (Freedman et al. 2014) so this approach is well‐suited for targeted enrichment and genomic sequencing in wolves. To avoid challenges with mapping reads, we excluded repetitive sequence regions like microsatellites, nested repeats, short interspersed elements, long interspersed elements, and other repeating regions that we identified using RepeatMasker 4.0.7 (Smit et al. 2015), Tandem Repeats Finder (Benson 1999), and WindowMasker (Morgulis et al. 2006). These excluded regions were already masked in the sequence file for the domestic dog genome or available on UCSC's TableBrowser (https://genome.ucsc.edu/cgi‐bin/hgTables). Our design also excluded exons (regions of the genome that code for proteins) and 100,000 base pairs (bp) flanking exons to avoid targeting SNPs under selection. We submitted our design to Roche (2024 F. Hoffmann‐La Roche Ltd) and after their proprietary screening and optimization to ensure efficient capture of target regions, the resulting probe set (SeqCap EZ Choice Probes) comprised 131,816 targets covering a total of 43,564,811 base pairs in the reference genome. The increased number of targets relative to the original capture design reflects the division of some targeted regions into one or more smaller fragments during Roche's proprietary screening and optimization process.

To further optimize the capture design, we prepared ten DNA samples for sequencing following the library preparation and capture protocol detailed below; then sequenced these samples on an Illumina MiSeq (v2 Nano 150 bp paired end reads) at the University of Montana Genomics Core (Missoula, MT, USA). Briefly, reads were processed through the removal of PCR duplicates step as detailed below in the section on Read Filtering, SNP Calling, and SNP Filtering. After removing PCR duplicates, we used Picard (v2.9.2, http://broadinstitute.github.io/picard/) CollectHsMetrics to assess fold‐enrichment and mean on‐target coverage. We then redesigned the capture, excluding all probes for which read coverage was three times or higher than the mean on‐target coverage for all targets with above 0× coverage. Many targets in the capture optimization sequencing returned 0× coverage due to a relatively low sequencing effort. We excluded the 0× coverage targets when calculating mean coverage to ensure that we did not remove more targets than necessary with the three times or higher mean coverage target filtering step. High coverage in the excluded regions is likely the result of reads from multiple paralogs mapping to a single region of the reference genome. These high coverage regions have lower mapping qualities and artificially inflated heterozygosity, so their removal from the capture design facilitated improved mapping quality scores, more accurate SNP genotype calls, and more accurate identification of ROH in subsequent analyses. The final probe set comprised 136,542 targets covering a total of 45,985,696 bp in the reference genome (Figure S2).

### 2.4 Library Preparation, Capture, and Sequencing

We prepared libraries according to Meyer and Kircher (2010) with the following modifications: we used a Covaris E220 to randomly fragment DNA to a mean size of 300 bp (125 μL sample volume in a microTUBE Snap‐Cap AFA Fiber, 140 W peak incident power, 10% duty factor, 200 cycles per burst, 55 s treatment time). For every post‐reaction solid phase reversible immobilization clean‐up step, we used Serapure beads (Faircloth and Glenn 2011). We used unique indexing oligos on both the P7 and P5 ends for each sample to avoid any cross‐sample contamination from tag‐switching during post‐pool PCR amplification steps.

We pooled 10 sample libraries (125 ng each) for each capture. For hybridization preparation, hybridization, recovery, and post‐capture amplification, we followed the protocol outlined in chapters five through seven in SeqCap EZ HyperCap Workflow User's Guide Version 2.1 (Roche), replacing the LM‐PCR oligos with HPLC‐purified primers IS5 and IS6 developed by Meyer and Kircher (2010). Thirty captured libraries were pooled in equimolar concentrations, and the pooled library was diluted to 10 nM total concentration and sent for sequencing on a single lane of a HiSeq 4000 (paired end 150 bp reads) at the University of Oregon Genomics and Cell Characterization Core Facility (GC3F, Eugene, OR, USA).

### 2.5 Bioinformatics

We used Trimmomatic v0.33 (Bolger et al. 2014) to remove adapters and discard reads shorter than 100 bp from demultiplexed reads to improve mapping quality scores. Unpaired reads were discarded to ensure high mapping quality scores, and we used PEAR v0.9.6 (Zhang et al. 2014) to assemble overlapping reads. Assembled and unassembled paired reads were aligned to canFam3.1 using BWA mem v0.7.15‐r1140 (Li 2013). We used Picard (http://broadinstitute.github.io/picard/) MergeSamFiles to merge SAM files for unassembled and assembled read pairs for each sample, MarkDuplicates to identify PCR duplicates, and SAMtools view (Li et al. 2009) to remove PCR duplicates. We then used Picard CollectHsMetrics to assess fold‐enrichment and mean on‐target coverage.

We used GATK v3.8 (McKenna et al. 2010) to identify SNPs. We used GATK to realign reads around indels and Picard (http://broadinstitute.github.io/picard/) to correct read mate information after realignment. We used GATK HaplotypeCaller to call raw SNPs for each individual within targeted regions and a 250 bp flanking region around each target. We used GATK GenotypeGVCFs to call SNPs using all samples, SelectVariants to filter out indels, and bcftools *view* (v1.16) to filter SNPs by allele frequency and u‐based *z*‐approximation from the Rank Sum Test for mapping qualities (MQRankSum; ‘INFO/AF > 0.01 and INFO/MQRankSum > −0.2’) resulting in a reference dataset of known SNP sites. We used GATK BaseRecalibrator to recalibrate base quality scores using the aforementioned reference dataset and the realigned, mate‐corrected BAM files. We called SNPs a final time using the recalibrated BAM files and GATK HaplotypeCaller and GenotypeGVCFs. Finally, we used VCFtools v0.1.16 (Danecek et al. 2011) to filter SNPs by minor allele frequency (−maf 0.05), minimum genotype quality (–minGQ 20), minimum mean coverage (−min‐meanDP 10), missingness (−max‐missing 0.85), Hardy–Weinberg proportions (−hwe 0.001), and to only retain biallelic sites (−min‐alleles 2 –max‐alleles 2) on autosomes 1–38, which resulted in 31,526 SNPs. We used VCFtools –relatedness 2 (Danecek et al. 2011) to screen for and remove duplicated samples.

### 2.6 Admixture and Population Structure

To evaluate population genetic structure and identify populations within the sampled region, we used 31,526 SNPs resulting from the above variant calling and filtering methods with ADMIXTURE v1.3.0 (Alexander and Lange 2011) to estimate individual ancestry from one to ten potential ancestral populations. The best supported number of ancestral populations was indicated by the minimum cross‐validation error (using 10‐fold cross‐validation) in our ADMIXTURE analysis. We used the highest proportion of individual ancestry to assign individuals to these putative populations. To evaluate genetic differentiation between the ADMIXTURE‐identified populations, we estimated pairwise *F*_ST_ using VCFtools. We used a principal components analysis (PCA) plot in EIGENSOFT v6.1.4 (Price et al. 2006) to visualize genetic differentiation between individuals.

### 2.7 Nucleotide Diversity and Inbreeding

To compare genetic variation and inbreeding of Southeast Alaskan wolves to another island wolf population of conservation concern, we included whole genome sequence data from a previous study of 11 wolves from Isle Royale National Park (IRNP) (Robinson et al. 2019). We obtained sequence reads from the 11 IRNP wolves (SRA Project PRJNA512209) and aligned them to canFam3.1 as described above. We converted the resulting SAM files to BAM format and then sorted BAM files using SAMTOOLS v1.15.1. We marked duplicate sequence reads using MarkDuplicates in GATK 4.2.0.0 and used BEDtools v2.29 (Quinlan and Hall 2010) to filter these BAM files so that only reads that overlap our sequence capture array targets remained. We then used Picard v2.23.9 to randomly remove sequence reads from each of the 11 IRNP wolves so that the genome‐wide average read depth for each IRNP wolf approximately equaled the average SNP read depth of the Southeast Alaskan wolves (9.7, after removing individuals with mean read depth ≤ 5). We used the GATK v4.2.0.0 HaplotypeCaller and GenotypeGVCFs for variant calling on IRNP and Southeast Alaskan wolves jointly. We used VCFtools 0.1.16 to remove indels, retain only biallelic SNPs within sequence capture target regions, and to remove SNPs that had average read depth > 24 in order to exclude SNPs likely to be in duplicated genomic regions (Kardos et al. 2018). This yielded 327,049 SNPs across all four ADMIXTURE‐identified populations (see Results Section 3.1). There were 157,306 SNPs in POW, 238,875 in the southern islands and mainland group (SE), 202,152 in the northwest group (NW), and 187,081 in IRNP.

We used estimated nucleotide diversity (π) in each ADMIXTURE‐identified SE Alaska population and IRNP based on genotype likelihoods with the program angsd () to account for genotype uncertainty (Korneliussen et al. 2014). Using the aligned sequence data from each population trimmed to include only sites covered by our capture array as input, we (1) calculated the site allele frequency posteriors assuming the reference allele was ancestral (‐doSaf 1 ‐GL 1), (2) used the posteriors from (1) to estimate the folded site‐frequency spectrum (SFS) (realSFS‐fold 1), (3) estimated site‐specific pair‐wise theta (realSFS saf2theta fold 1), (4) estimated pairwise theta (tP) across each chromosome (thetaStat do_stat), then (5) obtained the average genome‐wide π by dividing the summed chromosome‐specific estimates of tP by the total number of nucleotide sites in the genome with data.

We used SNP genotype likelihoods at all SNPs as input data for our analyses of inbreeding based on ROH (Khan et al. 2021; Kardos et al. 2023). The methods to detect ROH are described in detail in the Data S1. We identified ROH via analysis of genotype likelihoods as this approach uses information from all sequence reads at each SNP in every individual, including at loci with insufficient read depth for called genotypes to pass stringent filtering criteria (Nielsen et al. 2011; Kardos et al. 2023). However, we excluded individuals with average genome‐wide read depth < 5, as such low coverage can result in high error rates in the identification of ROH (Duntsch et al. 2021). This resulted in sample sizes for the inbreeding analysis of 14 (POW), 29 (SE), 6 (NW), and 11 (IRNP). We calculated *F*_ROH_ as the proportion of 38 autosomes in ROH ≥ 1 Mb (*F*_ROH ≥ 1Mb_), and ≥ 10 Mb (*F*_ROH ≥ 10Mb_). We repeated this analysis after equalizing the number of SNPs in each population by randomly pruning down to 157,308 SNPs in SE, NW, and IRNP to test whether variation in SNP density across populations strongly affected the results. To ensure the absence of spurious results, we tested for a relationship between *F*_ROH_ and sequence read depth using linear regressions using the lm () function in R (R Core Team 2021). Neither *F*_ROH ≥ 1Mb_nor *F*_ROH ≥ 10Mb_ had a significant relationship with sequence read depth (*p* = 0.34 and *p* = 0.1, respectively; Figure S3).

We generated bootstrap confidence intervals for average *F*_ROH_ in each population (Efron and Tibshirani 1994). For each of the four ADMIXTURE‐identified populations, we resampled *N F*_ROH_ values with replacement (*N* = the number of sampled individuals) 5000 times; each time recording the average of the resampled *F*_ROH_ values. The 95% non‐parametric bootstrap confidence interval was determined as the 0.025 and 0.975 quantiles of the 5000 means.

We analyzed the length distributions of ROH to evaluate inbreeding in each wolf population due to recent versus deep historical demographic history. The breaking up of haplotypes by recombination during meiosis means that very long ROH are likely to arise from recent common ancestors of parents, whereas short ROH likely originate from common ancestors in deeper history. The number of generations (*g*) back to the ancestor where an ROH arises can be estimated by solving for *g* in *l* = 100/2 *g* cM, where *l* = the genetic map length of an ROH in centiMorgans, under the assumption that the coalescent pattern is constant across an entire ROH (Thompson 2013). A high abundance of ROH with similar estimated *g* suggests a relatively small effective population size (*N*_e_) during that time compared to populations with less frequent such ROH (Kirin et al. 2010). We estimated *g* for each ROH in every individual, assuming recombination rates from a domestic dog linkage map (Campbell et al. 2016). We then calculated the average Mb in ROH within each of six ranges of *g* (< 10, 10–20, 20–30, 30–40, 40–50, > 50 generations) for each population as a metric of inbreeding arising from ancestors in different historical time periods.

## 3 Results

### 3.1 Admixture and Population Structure

**FIGURE 2:**
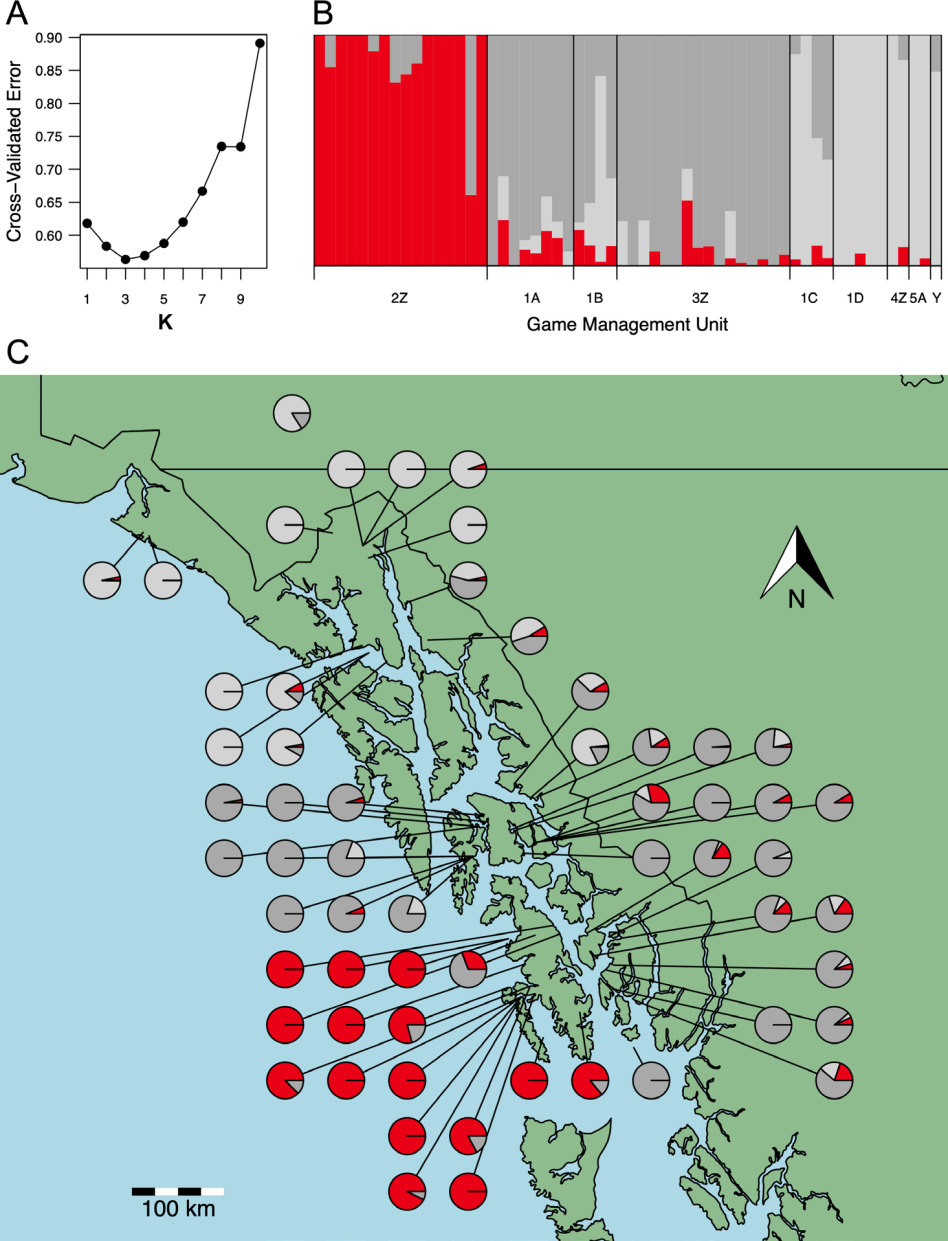
Summary of ADMIXTURE analysis results to determine individual wolf population assignments for each Alexander Archipelago wolf sampled in Southeast Alaska and Yukon Territory. (A) Cross‐validated errors for one through 10 putative populations. (B) Proportion of ancestry for wolves. (C) Spatial distribution of the estimates of individual ancestry proportions shown in (A). Ancestry proportions shown assume three ancestral populations as indicated by the lowest cross‐validation error (B).

In our ADMIXTURE analysis of one through 10 potential ancestral populations, we found that three ancestral populations were best supported (Figure 2A and Figure S4): (1) Prince of Wales Island and the adjacent GMU 2 islands Dall, Long, and Suemez (POW, *n* = 15); (2) the southern inside islands (Kuiu, Kupreanof, Mitkof, Revilla, and Duke) and southern mainland (southeastern group [SE], *n* = 29); and (3) the northern mainland west of Lynn Canal, Pleasant Island, and the Yukon Territory (northwest group [NW], *n* = 14). One of the wolves sampled on POW had majority assignment to the SE group and therefore likely has a recent ancestor that immigrated from the SE population to POW, highlighting the possibility of natural migration across water bodies (Figure 2B,C). No wolves sampled outside of POW had majority ancestry assignment to the POW group, indicating that migration observed in this study was unidirectional. The mainland appeared to be a landscape corridor facilitating admixture, as one wolf sampled in the southern mainland had majority ancestry assignment to the NW group, and 2 wolves sampled in the northern mainland had majority or nearly majority assignment to the SE group (Figure 2A,C).

**FIGURE 3:**
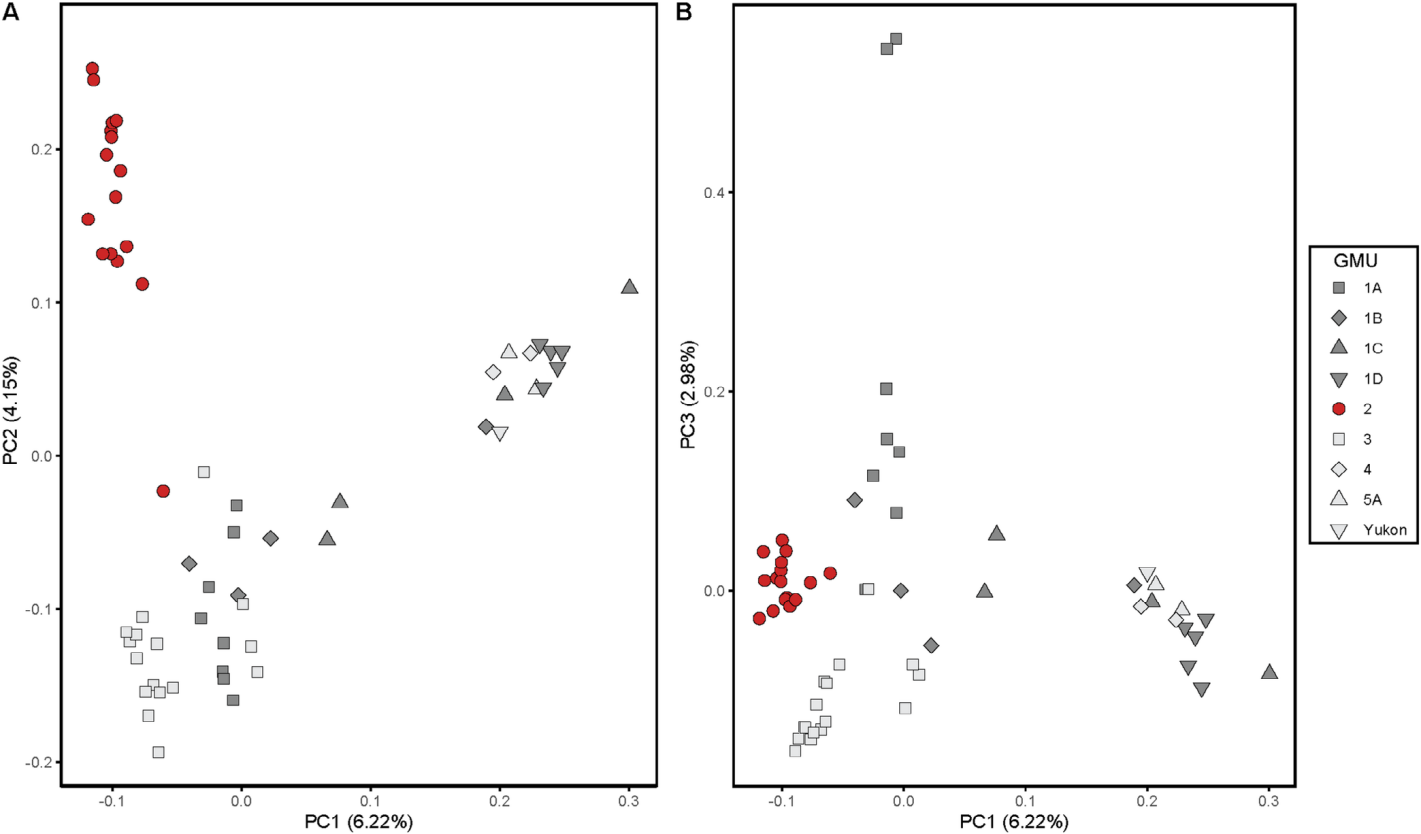
Principal components analysis of 58 wolves sampled in Southeast Alaska, USA and the Yukon Territory, Canada. Color and symbol for each sample correspond to Game Management Units (GMUs, shown in Figure 1), and one sample from Yukon, as indicated in the legend. Wolves do not inhabit islands in Game Management Unit (GMU) 4Z, with the exception of Pleasant Island, which was administratively changed to GMU 1C in 2022. (A) PC2 versus PC1, (B) PC3 versus PC1.

We used PCA to describe the first three principal components (PCs) in 31,526 SNPs (Figure 3) for all 58 individuals. Reflecting the results observed in the ADMIXTURE analysis, samples grouped into three clusters, with POW comprising one cluster (red circles in Figure 3A). The other two clusters were comprised of a mixture of samples from the remaining GMUs. The one sample collected in the Yukon Territory clustered with samples from northern Lynn Canal, Pleasant Island, and the Yakutat Forelands (GMUs 1C, 1D, and 5A). The POW wolves were not distinct from other wolves on PC1 or PC3, which explained 6.22% and 2.98% of the genetic variation observed in all samples, respectively (Figure 3B). Examination of the relationship between genetic divergence and individuals (*n* = 2) with a high missingness profile (> 0.85 proportion missing data) showed that they were not more divergent than other individuals in the group to which they were assigned (Data S1). The POW wolves separated from other wolves on PC2, which explained 4.15% of the genetic variation observed across all samples (Figure 3A). Pairwise estimates of *F*_ST_ were 0.16 between POW and SE, 0.23 between POW and NW, and 0.20 between the SE and NW groups.

### 3.2 Nucleotide Diversity and Inbreeding

**FIGURE 4:**
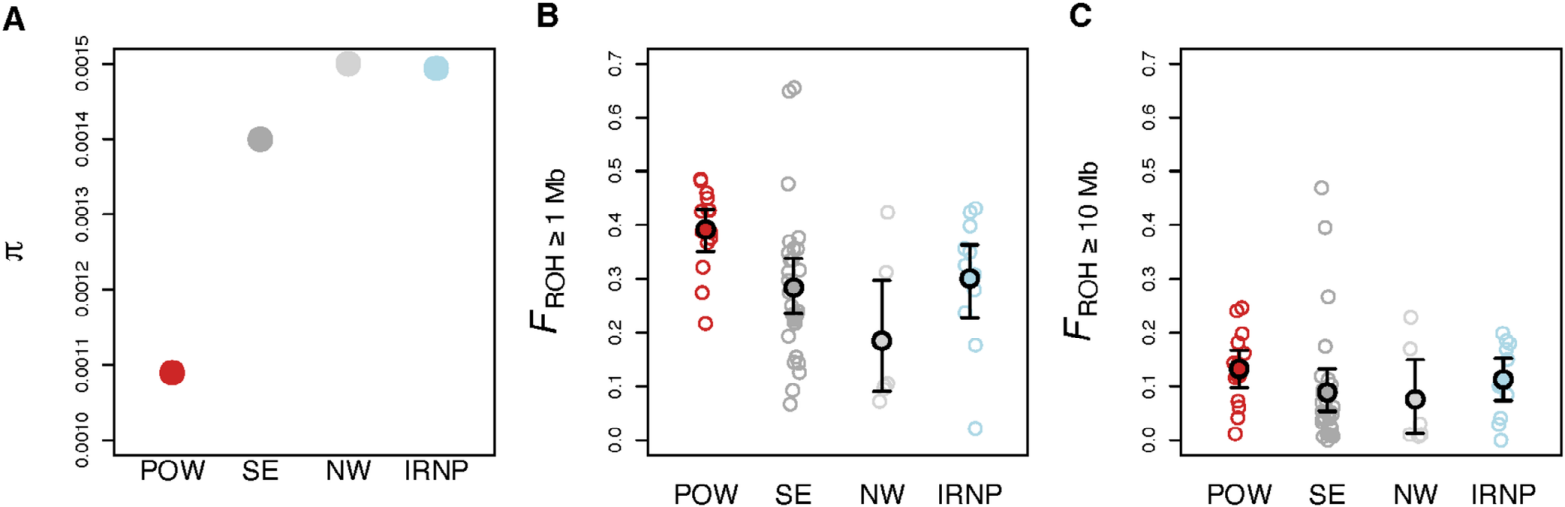
Nucleotide diversity (π) and genomic inbreeding coefficients for wolves in Admixture‐defined populations (Prince of Wales Island [POW; *n* = 14], the southern mainland and inside islands [SE; *n* = 29], and the northern mainland and the Yukon Territory, Canada [NW; *n* = 6]) and Isle Royale National Park, USA (IRNP; *n* = 11; Robinson et al. 2019) was included for comparison. (A) Nucleotide diversity was calculated considering all regions in the wolf genome covered by our custom sequence capture array. (B, C) *F*_ROH_ was measured using ROH ≥ 1 Mb (B), and ≥ 10 Mb (C). Filled points in B, C represent the average and bars represent 95% bootstrap confidence intervals.

The POW population had the lowest *π* and highest inbreeding among the four populations we evaluated. Estimated *π* for POW wolves (0.00109) was ≥ 20% lower than in IRNP (0.00149), SE (0.00140), and NW (0.00150) wolves (Figure 4A). The POW group had the highest inbreeding among the three ADMIXTURE‐defined groups of Alaska wolves and wolves in IRNP. The highest mean *F*_ROH ≥ 1Mb_ was in the POW group (mean = 0.39, range = 0.22–0.49), followed by IRNP (mean = 0.30, range = 0.02–0.43), followed by SE (mean = 0.28, range = 0.07–0.66), then NW (mean = 0.18, range = 0.07–0.42; Figure 4B). Long ROH (*F*_ROH ≥ 10Mb_) indicative of inbreeding due to recent ancestors were more abundant in IRNP and POW wolves than in the SE and NW groups, but these differences were not substantial (Figure 4C). The highest mean *F*_ROH ≥ 10Mb_ was observed in the POW group (mean = 0.13, range = 0.01–0.25), followed by the IRNP (mean = 0.11, range = 0.00–0.20), followed by the SE group (mean = 0.09, range = 0.00–0.47), then the NW group (mean = 0.08, range = 0.01–0.23). Repeating the *F*_ROH_ analysis with equalized SNP numbers across populations did not substantively affect the results (Figure S5).

**FIGURE 5:**
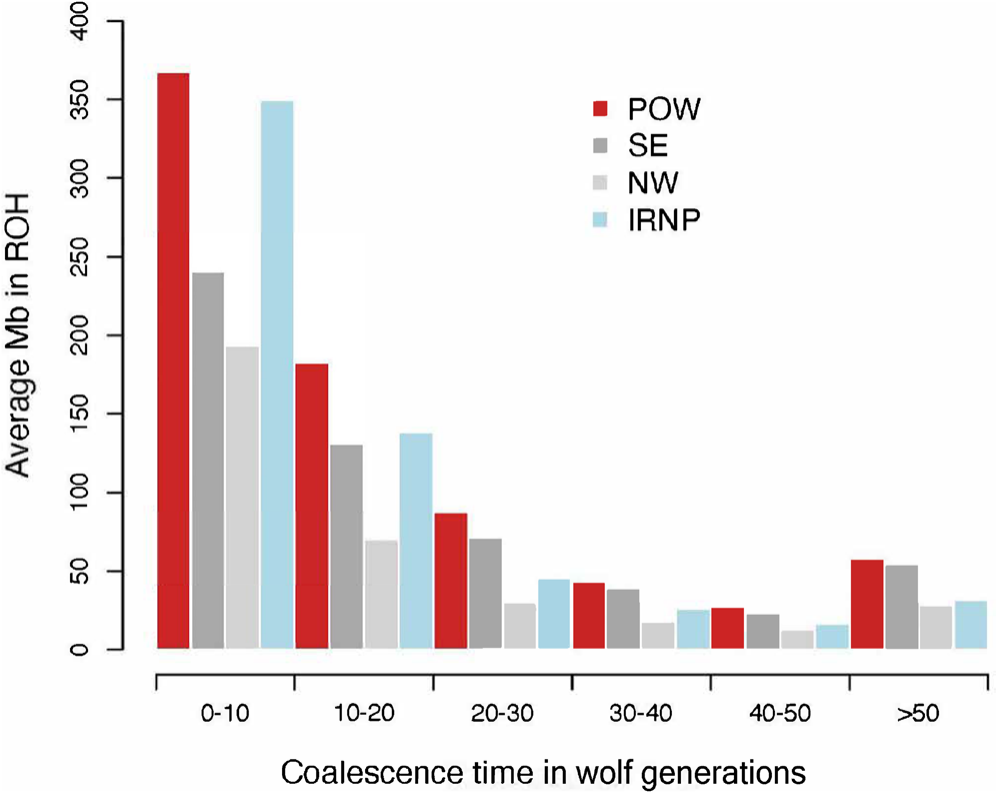
Inbreeding of wolves arising from ancestors in different estimated historical time periods. The values on the y‐axis are the average Mb covered in runs of homozygosity (ROH) with given estimated coalescent time (wolf generations) as indicated on the x‐axis. The three populations in our Southeast Alaska, USA, study area identified with ADMIXTURE (Prince of Wales Island [POW; *n* = 14], the southern mainland and inside islands [SE; *n* = 29], and the northern mainland and the Yukon Territory, Canada [NW; *n* = 6]) are shown, and Isle Royale National Park, USA (IRNP; *n* = 11; Robinson et al. 2019) was included for comparison.

The distributions of estimated coalescent times for haplotypes comprising ROH suggest that POW wolves had the highest inbreeding due to small population size both recently and in deep history (Figure 5). Specifically, POW wolves had the most ROH arising from ancestors in the last 10 generations, and in all of the deeper historical timeframe bins (Figure 5). Wolves in the IRNP group had an abundance of ROH with coalescent time < 10 generations that was only slightly lower than that of POW (Figure 5). However, IRNP had fewer average Mb in ROH with deep historical coalescent times compared to both SE and POW Alaska wolves, which may reflect the recent colonization of Isle Royale and large *Ne* of the mainland source population (Hedrick et al. 2014).

## 4 Discussion

The objective of this work was to evaluate population structure and individual inbreeding in Alexander Archipelago wolves in Southeast Alaska, with a particular emphasis on the Prince of Wales Island complex (POW) because it is geographically isolated, has previously been identified as a population of conservation concern (U.S. Fish and Wildlife Service 2023), and the maintenance of the population is a management priority (Article 8, Section 4 of the Alaska Constitution; Wolf Technical Committee 2017). We developed a cost‐effective and robust SNP genotyping method to evaluate population genomic structure and individual inbreeding in wolves to address these questions. Relative to other populations in Southeast Alaska and wolves from Isle Royale National Park (IRNP), wolves on POW had low genetic variation and high inbreeding estimates, suggesting that future work to understand whether the population is exhibiting inbreeding depression is necessary to ensure appropriate management actions and long‐term population persistence.

### 4.1 Admixture and Population Structure

Our population genetic structure analyses revealed three distinct populations within the sampling area. This result is consistent with geographic features (e.g., islands, mountains, fjords) contributing to habitat fragmentation and increased resistance to dispersal in some areas of the archipelago, and other features (e.g., the mainland, low elevation river corridors, shallow and narrow water channels) serving as regions of genetic connectivity. The POW group was most readily distinguished from wolves elsewhere in Southeast Alaska by PCA and ADMIXTURE analyses consistent with previous research (Weckworth et al. 2005; Cronin et al. 2015). The southeast group (SE) encompassed five major islands and the southern mainland. Unlike the POW island complex, which is separated from other island groups by wide expanses of water such as Clarence Strait (Figure 1), the islands in the SE group have several narrow water passages < 1 km that are shallower and have weaker current and therefore would present less of a barrier to wolves migrating between islands and the mainland. Water barriers of 2–7 km have been demonstrated to limit gene flow of brown bears between islands in Southeast Alaska (Paetkau et al. 1998) and similar patterns could be expected for wolves. The northwest group (NW) consisted entirely of northern mainland wolves and included the Yukon wolf, likely connected to the coastal Alaskan population by the several river valleys that extend into the interior of the Yukon Territory and British Columbia. The mainland of the southeast Alaskan panhandle appears to provide an area of geographic connectivity as admixture of the SE and NW populations was evident in the individual ancestry assignments (Figure 2B,C).

Although wolves have not been documented moving between POW and other GMUs, including islands and mainland units, genetic recapture data of wolves sampled on the islands of Snow Pass to the northeast of POW and on POW and other islands in the complex indicate dispersal has occurred (Roffler et al. 2024). The islands of Snow Pass and Zarembo could provide a potential pathway for wolves to migrate to or from the mainland or other southern island groups by swimming between the islands (longest straight‐line swim = 2.7 km; Figure 1). This stepping‐stone corridor was possibly the route taken by the one immigrant or offspring of an immigrant wolf from the SE group we identified on POW using genomic data in this study, which provides evidence that wolves can move between other GMUs (both island and mainland units) and POW. This immigrant wolf sired two litters on POW (Roffler et al. 2018) providing further evidence of gene flow. However, outside of this instance, the frequency of immigration events is unknown, as is the probability of these immigrants settling and reproducing with local POW wolves. Our results show that there are sufficient barriers to movement between POW and other parts of Southeast Alaska to inhibit all but infrequent migration of wolves among geographic regions, resulting in substantial genetic differentiation and genetic isolation of POW wolves.

The dispersal distances documented within the POW population through previous genetic recapture efforts were short in general (average 41.9 km, SD = 23.7 km) and occurred throughout Prince of Wales Island and other islands in the POW complex (Roffler et al. 2024). The straight‐line dispersal distances were likely constrained by the geography of the relatively isolated island system and could result in a higher density of related wolves, making it more difficult to find an unrelated mate and rendering inbreeding avoidance strategies less effective (Viluma et al. 2022). Unlike other continental wolf populations that have substantial genetic connectivity to adjacent populations facilitated by continuous habitat and long‐distance dispersal capabilities, POW wolves remain restricted by their island geography, which is likely contributing to higher rates of breeding with close relatives.

### 4.2 Nucleotide Diversity and Inbreeding

Wolves in the POW group exhibited the lowest π and highest *F*_ROH ≥ 1Mb_ and *F*_ROH ≥ 10Mb_ when compared to the two other Southeast Alaska populations (SE and NW) and wolves from IRNP. The most striking result in our *F*_ROH_ analyses was that despite the current relatively large annual population census sizes of POW wolves indicating a stable or growing population and consistent reproduction and juvenile survival (Dorendorf 2022; Figure S1B), they have experienced very high levels of inbreeding likely due to a small recent population size in the past 10 generations. Wolf generations are estimated at between 4.2 and 4.7 years (Mech et al. 2016), therefore this population bottleneck corresponds to approximately 1970. Although wolves in the POW population had the highest levels of inbreeding, wolves in the southeast (SE) group also demonstrated relatively high inbreeding measured by short *F*_ROH_ (≥ 1 Mb), comparable to the extent of inbreeding observed in IRNP wolves. Wolves in the northwest (NW) group had the lowest *F*_ROH_ estimates, indicating that this population is relatively large and/or more genetically connected with nearby populations. Although POW and IRNP had the highest proportion of Mb in ROH, all three Southeast Alaska populations showed an increase during the last 0–10 generations indicating an overall genetic bottleneck (Figure 5).

It is important to note that POW wolf samples included in this current analysis were collected mostly during 2015 (Table S1), which is too soon after the lowest population estimate in 2014 (*n* = 89; 95% CI = 50–159) to capture any effect this decline in abundance may have had on individual genomic inbreeding. Therefore, the temporary low population size in 2014 could have resulted in increased mating events between related individuals in subsequent years; it is therefore possible that POW wolves currently have higher *F*_ROH_ inbreeding coefficients than estimated by the data and results presented here.

Our analysis of coalescence for haplotypes in ROH revealed additional similarities between POW wolves and IRNP wolves, with both populations demonstrating similar extents of genomic inbreeding as the result of mating events between close relatives within the last 10 generations. These results are notable because the IRNP population was founded by just two or three individuals in the late 1940s (Wayne et al. 1991); peaked at 50 individuals in 1980 (Peterson et al. 2014); and received only one immigrant in 1997 (Adams et al. 2011) before successful reproduction stopped in 2014 as the result of severe inbreeding depression (Peterson and Vucetich 2017), and the population went functionally extinct (Hedrick et al. 2019). The similarities in *F*_ROH_ and ROH coalescent time between POW and IRNP indicate that POW wolves have experienced substantial inbreeding in recent years; and due to limited immigration from other populations, are at heightened risk for exhibiting inbreeding depression.

Wolf populations with low genetic diversity and high individual inbreeding estimates have exhibited inbreeding depression, including IRNP wolves (Räikkönen et al. 2009; Robinson et al. 2019), Mexican wolves (Fredrickson et al. 2007; Clement et al. 2024), and Scandinavian wolves (Liberg et al. 2005; Smeds and Ellegren 2023). Inbreeding depression in these populations has been documented via reduced litter size, reduced juvenile survival, and morphological anomalies (Salado et al. 2024 and citations therein). Between 2013 and 2017, at least three wolves on POW have been observed with shortened tails, but the causes of these deformities are unknown, and they could be either the result of inbreeding depression similar to the defects observed in IRNP and Scandinavian wolves (e.g., Räikkönen et al. 2009; Hedrick et al. 2016; Räikkönen et al. 2013) or from physical trauma. These anecdotal observations are not sufficient to either confirm or reject that wolves in Southeast Alaska are currently exhibiting inbreeding depression.

Inbreeding depression is expected to occur in all diploid populations due to the ubiquity of deleterious mutations and genetic drift (Lacy 1997; Kardos et al. 2021), including in populations that have been small and isolated for long periods (Stoffel et al. 2021; Kardos et al. 2023). Inbreeding increases homozygosity and results in the expression of deleterious, partially recessive alleles as well as reductions in the frequency of loci that are advantageous as heterozygotes. In some small populations that have been isolated over the long term, inbreeding may facilitate the purging of very highly deleterious alleles via natural selection as previously reported in island foxes and island dingoes (Robinson et al. 2018; Leon‐Apodaca et al. 2024; but see Ralls et al. 2020 for a critique on this perspective). However, purging is not expected to eliminate all deleterious alleles (Hedrick and Garcia‐Dorado 2016; Dussex et al. 2021; Khan et al. 2021), and consistently restricted population size means deleterious alleles are expected to reach a high frequency or fixation, resulting in decreased mean fitness in a population (Lynch et al. 1995). As demonstrated by the now extinct Sierra Morena wolves (the southernmost population of the Iberian wolf range), historical persistence of a small, isolated population should not be considered evidence of viability into the future (Gómez‐Sánchez et al. 2018).

Wolves in Southeast Alaska diverged from nearby populations in interior Alaska and interior British Columbia approximately 12,000 to 16,000 years ago and have consistently demonstrated a low effective population size that has been declining for the past approximately 600 years (Nowak and Paradiso 1983; Nowak 1995; Pacheco et al. 2022). Low genetic diversity and high inbreeding coefficients reflect the declining effective population size, which has been particularly exacerbated during the past 280 years (Pacheco et al. 2022). This long‐term isolation and geographic restriction of wolves in Southeast Alaska and in POW in particular have maintained low population sizes for thousands of years and may have facilitated the purging of deleterious recessive alleles. On the other hand, this historical pattern of a lack of gene flow leading to low genetic variation and very high individual inbreeding could result in inbreeding depression. It is important to note that despite the lack of evidence, no explicit investigations to detect inbreeding depression as a result of the genetic bottleneck and isolation have been conducted in POW wolves. Additional work is necessary to understand whether the observed low genetic variation and high individual inbreeding represent significant risks to the long‐term persistence of populations in this study and whether management actions like translocation of individuals would be beneficial.

Translocations or assisted migration, introducing captive animals, and providing foster offspring are among the tools that managers may consider to bolster genetic diversity in inbred wolf populations (Harding et al. 2016; Hervey et al. 2021; Clement et al. 2024). Gene flow from other populations can offset the negative effects of inbreeding in small, isolated populations; recommendations include maintaining gene flow of > 1 individual per generation (Laikre et al. 2016). However, as shown in the IRNP population, infrequent immigration of highly reproductively successful individuals into an extremely small population can result in much of an immigrant's genome rapidly sweeping to high frequency, ultimately exacerbating inbreeding and inbreeding depression (Hedrick et al. 2019). Scandinavian wolves also present a case where immigration temporarily mitigated inbreeding depression in a small and isolated population (Åkesson et al. 2016). The wolf population in Sweden was established by two individuals in 1983 and remained small and highly inbred until immigration in 1991 and 2007, which led to higher reproductive success in inbred wolves and population growth (Åkesson et al. 2016). However, those effects were short‐lived as heterozygosity decreased after a couple of years, and the immigrants also delivered new deleterious alleles to the population (Smeds and Ellegren 2023). Therefore, translocation efforts must be well researched and designed to provide regular inputs of genetic diversity into the receiving population while avoiding the introduction of deleterious alleles or counterproductive genetic sweeps.

## 5 Conclusions and Future Work

The low genetic variation and high individual inbreeding estimates in wolves on POW highlight the need to understand whether the population is exhibiting inbreeding depression before proposing and implementing management actions. Although we did not measure inbreeding depression in this study, this should be a priority for future research because the demographic consequences of inbreeding depression are difficult to predict without directly measuring these effects. However, measuring inbreeding depression is challenging in wild populations (Kardos et al. 2016), particularly those that are cryptic and rare. Additionally, estimating the reduction in fitness due to inbreeding depression is a formidable task in wildlife populations, as this requires significant sampling of difficult‐to‐acquire fitness measurements (e.g., litter size, pup survival) over long periods of time (Clement et al. 2024). Ideally, future research would assess the relationship of breeding success and survival to individual heterozygosity and especially differences in fitness among known immigrants, their offspring, and resident inbred wolves. Alternatively, future research could use whole genome sequencing to estimate the accumulation of deleterious genetic variants, which could lead to reductions in fitness (Bertorelle et al. 2022). Furthermore, simulation studies to predict the effects of potential wolf relocations, outbreeding, or fostering on increasing heterozygosity and reducing levels of inbreeding would be beneficial for managing viable populations of wolves in Southeast Alaska (Harding et al. 2016; Hervey et al. 2021).

This study presents a valuable use of genomic capture data to further our understanding of the status of Alexander Archipelago wolves on POW and in Southeast Alaska. Our results provide a solid foundation to promote and continue monitoring efforts to understand genetic structure and inbreeding in a population of conservation concern.

## Conflicts of Interest

The authors declare no conflicts of interest.

## Supporting information


**Appendix S1:** eva70144‐sup‐0001‐AppendixS1.docx.

## Data Availability

Raw DNA sequence reads generated in this study were submitted to the National Center for Biotechnology Information (NCBI) Sequence Read Archive (SRA) database under Accession Project no. PRJNA1194631. Wolf capture targets are available from Dryad https://doi.org/10.5061/dryad.x3ffbg7wh.
