# Supplementary material for: Genomic Analysis Reveals Inbreeding in an Island Population of Alexander Archipelago Wolves
Source: Evol Appl. 2025 Aug 12;18(8):e70144. doi: 10.1111/eva.70144 (PMC12340708; doi:10.1111/eva.70144)
Supplement: Supplementary file 1 — Appendix S1: eva70144‐sup‐0001‐AppendixS1.docx. [file EVA-18-e70144-s001.docx]

**SUPPLEMENTARY MATERIALS**

**Detecting Runs of Homozygosity**

To identify ROH, we used a sliding window-based analysis method that uses genotype likelihoods rather than called genotypes as input (Khan et al. 2021, Kardos et al. 2023). This approach evaluates the probability of each observed genotype under the assumption that the genotype is identical-by-descent, and the probability of the observed genotype under the assumption that the locus is not identical-by-descent (probabilities calculated using equations from (Wang et al. 2009). A logarithm of odds (LOD) score for each window is calculated by summing across the log_10_ of the ratio of these probabilities across all loci in the window (Kardos et al. 2018). A Gaussian kernel density function to visualize LOD scores for all individuals across all genomic windows, following Pemberton et al. (2012). The resulting curve was bimodal, with windows in runs of homozygosity comprising the right mode, and loci not in runs of homozygosity comprising the left mode in the distribution of LOD scores. A threshold LOD score is identified, above which a window is considered as identical-by-descent, as the minimum of the Gaussian density curve between the two modes. Overlapping identical-by-descent windows are joined to form inferred ROH, and *F*_ROH_ is calculated as the fraction of the genome in ROH.

**Assessing Genetic Divergence and the Effect of Missing Data**

We assessed the relationship with the missingness profiles and individual wolf genetic divergence by examining the PCA plots. The PCA results showed the individuals with the highest degree of missingness plotted in the center of their respective groupings, and indicated they were not more divergent than other samples within the population with which they were grouped. We also assessed the relationship with the missingness profiles and genetic divergence by examining the ADMIXTURE results. Only two individuals had > 0.85 proportion missing data, and those individuals had a high proportion (>0.75) of individual ancestry in their assigned populations. Both of these individuals were sampled within their respective population geographies and were not flagged as recent immigrants. Therefore, the individuals in our dataset were not genetically divergent due to higher levels of missing data.

**SUPPLEMENTARY FIGURES**

**Figure S1.** (**A**) Number of wolves harvested annually in GMU 2, regulatory 1985–2023. Black bars indicate years the wolf harvest season was closed early by emergency order. (**B**) Annual fall (October-December) population estimates and 95% confidence intervals for Alexander Archipelago wolves (*Canis lupus ligoni*) in Game Management Unit (GMU) 2, 2013–2022, Alaska, USA.


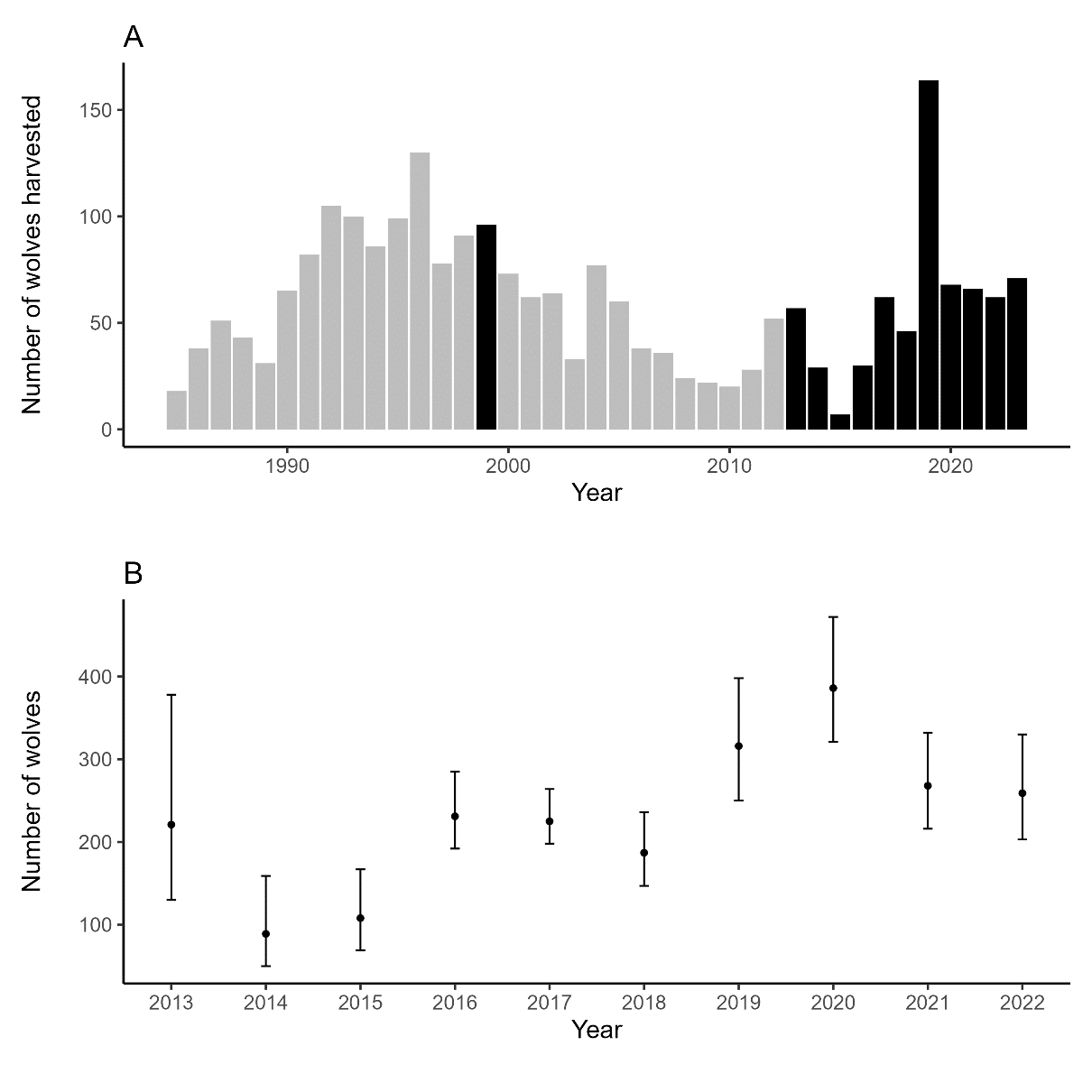


**Figure S2.** Distribution of sequence capture baits across 38 autosomes in the dog reference genome, CanFam 3.1.


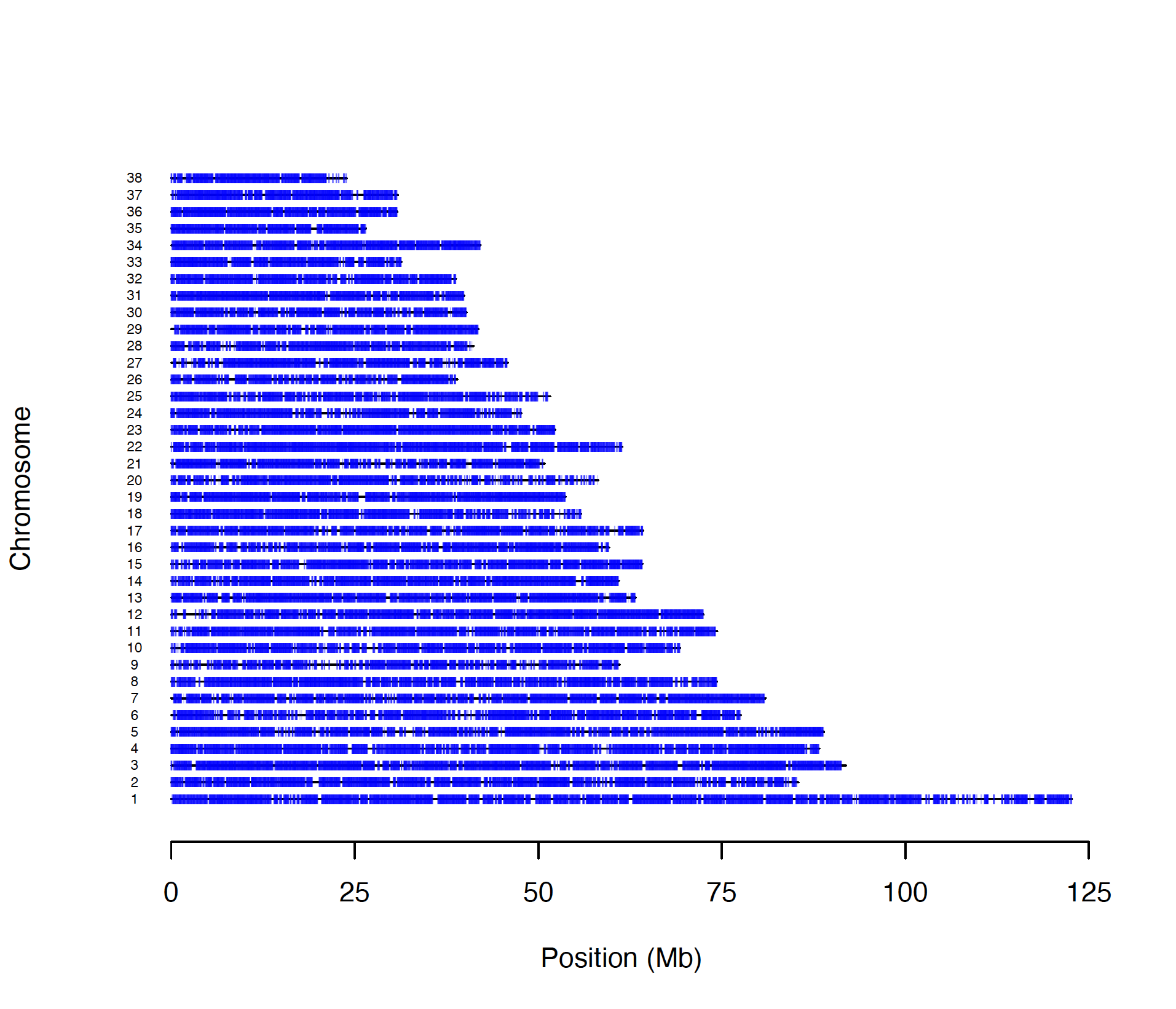


**Figure S3.** Non-significant relationship between F_ROH ≥ 1Mb_ or F_ROH ≥ 10Mb_ and sequence read depth.


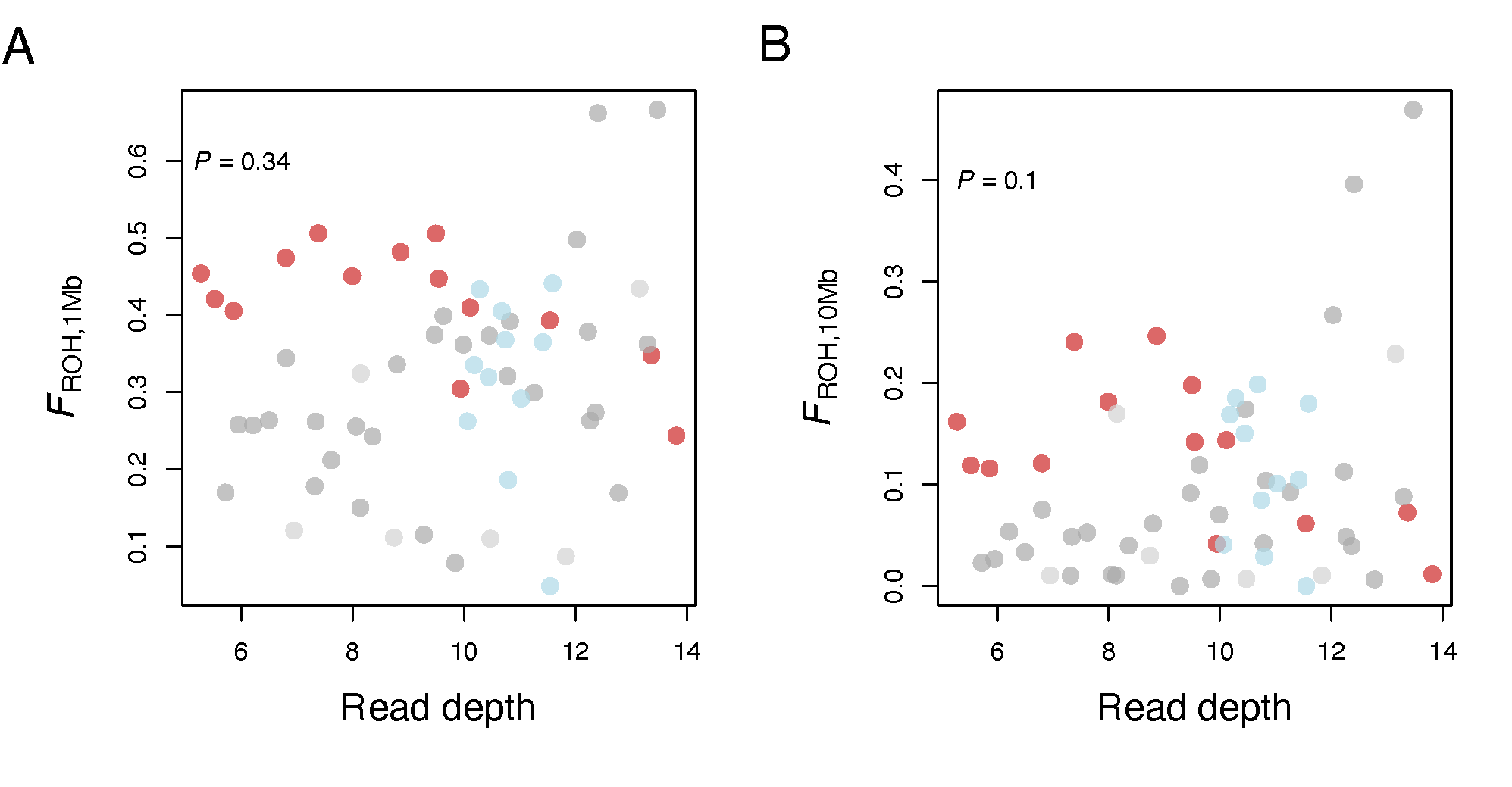


**Figure S4.** Seven ADMIXTURE plots showing proportion ancestry assignment for each Alexander Archipelago wolf (*Canis lupus ligoni*) assuming two through eight populations. Each vertical bar represents one individual, and colored sections of each bar represent proportion ancestry for that individual as identified by ADMIXTURE. ADF&G Game Management Units labeled at base of each ADMIXTURE plot, with the exception of “Y” which represents one sample collected in Yukon Territory, Canada. Wolves do not inhabit islands in Game Management Unit (GMU) 4Z, with the exception of Pleasant Island which was administratively changed to GMU 1C in 2022.


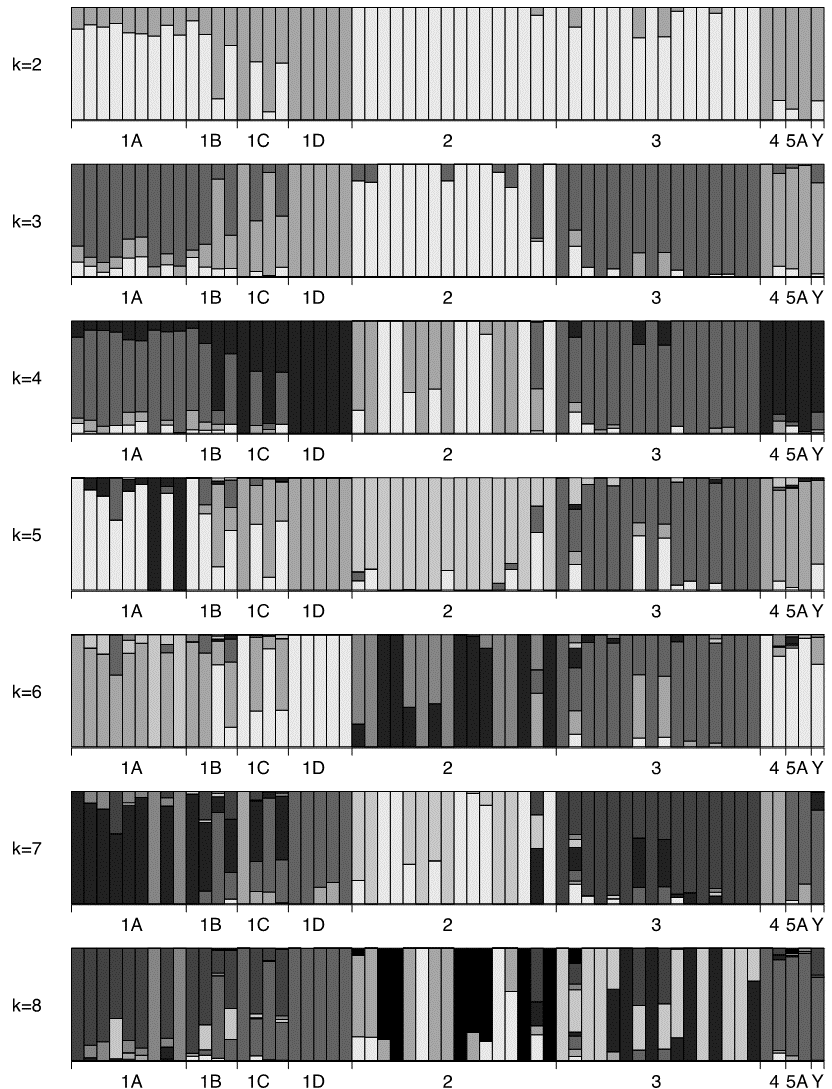


**Figure S5.** Genomic estimates of inbreeding for Alexander Archipelago (POW, SE, and NW) and Isle Royale National Park (IRNP) wolves. *F*_ROH_ was measured using runs of homozygosity (ROH) > 1Mb (A), and > 10 Mb (B) after pruning the SNPs down to 157,306 loci for SE, NW, and IRNP. Filled points represent the average and bars in represent 95% bootstrap confidence intervals.


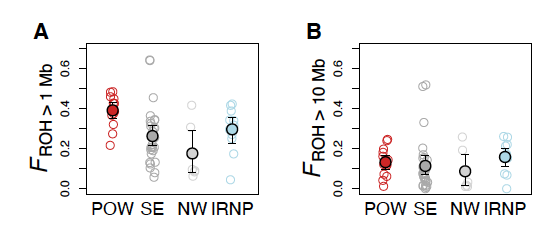


**SUPPLEMENTARY TABLES**

**Table S1.** Collection years of wolf samples by Game Management Unit (GMU) in Southeast Alaska, USA and the Yukon Territory, Canada. Wolves do not inhabit islands in Game Management Unit (GMU) 4Z, with the exception of Pleasant Island which was administratively changed to GMU 1C in 2022.

| **GMU** | **2002** | **2003** | **2004** | **2005** | **2006** | **2007** | **2008** | **2009** | **2010** | **2011** | **2012** | **2013** | **2014** | **2015** | **2016** | **Unknown** | **Total** |
| --- | --- | --- | --- | --- | --- | --- | --- | --- | --- | --- | --- | --- | --- | --- | --- | --- | --- |
| **1A** |  | 1 | 3 |  |  |  |  |  |  |  |  |  |  | 4 |  |  | 8 |
| **1B** |  | 1 |  |  | 1 |  |  |  |  |  |  |  |  | 1 |  | 1 | 4 |
| **1C** |  |  |  |  |  |  |  |  | 1 |  | 1 |  |  | 1 | 1 |  | 4 |
| **1D** | 1 | 3 | 1 |  |  |  |  |  |  |  |  |  |  |  |  |  | 5 |
| **2** |  |  | 1 |  |  |  |  |  |  |  |  |  |  | 15 |  |  | 16 |
| **3** |  |  |  |  |  | 2 |  |  |  |  |  | 1 | 1 |  | 12 |  | 16 |
| **4** |  |  |  |  |  |  |  |  |  |  |  |  |  |  | 2 |  | 2 |
| **5A** |  |  |  | 2 |  |  |  |  |  |  |  |  |  |  |  |  | 2 |
| **Yukon** |  |  |  |  |  |  |  |  |  | 1 |  |  |  |  |  |  | 1 |
| **Total** | 1 | 5 | 5 | 2 | 1 | 2 | 0 | 0 | 1 | 1 | 1 | 1 | 1 | 21 | 15 | 1 | 58 |

**REFERENCES**

Kardos, M., M. Åkesson, T. Fountain, Ø. Flagstad, O. Liberg, P. Olason, H. Sand, P. Wabakken, C. Wikenros, and H. Ellegren. 2018. Genomic consequences of intensive inbreeding in an isolated wolf population Nature ecology & evolution 2:124-131.

Kardos, M., Y. Zhang, K. M. Parsons, A. Yunga, H. Kang, X. Xu, X. Liu, C. O. Matkin, P. Zhang, E. J. Ward, B. M. Hanson, C. Emmons, M. J. Ford, G. Fan, and S. Li. 2023. Inbreeding depression explains killer whale population dynamics. Nature ecology & evolution 7:675-686.

Khan, A., K. Patel, H. Shukla, A. Viswanathan, T. van der Valk, U. Borthakur, P. Nigam, A. Zachariah, J. Yadavendradev, M. Kardos, and U. Ramakrishnan. 2021. Genomic evidence for inbreeding depression and purging of deleterious genetic variation in Indian tigers. Proceedings of the National Academty of Sciences 118:e2023018118.

Pemberton, T. J., D. Absher, M. W. Feldman, R. M. Myers, N. A. Rosenberg, and J. Z. Li. 2012. Genomic patterns of homozygosity in worldwide human populations. The American Journal of Human Genetics **91**:275-292.

Wang, S., C. Haynes, F. Barany, and J. Ott. 2009. Genome‐wide autozygosity mapping in human populations. Genetic epidemiology **33**:172-180.
